# The impact of gravidity, symptomatology and timing of infection on placental malaria

**DOI:** 10.1186/s12936-020-03297-3

**Published:** 2020-06-24

**Authors:** Erin E. Tran, Morgan L. Cheeks, Abel Kakuru, Mary K. Muhindo, Paul Natureeba, Miriam Nakalembe, John Ategeka, Patience Nayebare, Moses Kamya, Diane Havlir, Margaret E. Feeney, Grant Dorsey, Stephanie L. Gaw

**Affiliations:** 1 Division of Maternal Fetal Medicine, Department of Obstetrics Gynecology & Reproductive Sciences, University of California San Francisco, 513 Parnassus Ave. HSE16, Box 0556, San Francisco, CA 94143 USA; 2 Present Address: Division of Maternal Fetal Medicine, Department of Obstetrics & Gynecology, NorthShore University HealthSystem, Evanston, IL USA; 3 Infectious Diseases Research Collaboration, Kampala, Uganda; 4 Makerere University College of Health Sciences, Kampala, Uganda; 5 Division of Infectious Diseases, Department of Medicine, University of California San Francisco, San Francisco, CA USA; 6 Division of Pediatric Infectious Diseases, Department of Pediatrics, University of California San Francisco, San Francisco, CA USA

**Keywords:** Perinatal outcome, Infectious disease, Pregnancy, Global health, Primigravid, Africa, Obstetrics, *Plasmodium falciparum*

## Abstract

**Background:**

Placental malaria is associated with increased risk of adverse perinatal outcomes. While primigravidity has been reported as a risk factor for placental malaria, little is known regarding the relationship between gravidity, symptomatology and timing of *Plasmodium falciparum* infection and the development of placental malaria.

**Methods:**

The aim of this study was to investigate the relationship between the development of placental malaria and gravidity, timing of infection, and presence of symptoms. This is a secondary analysis of data from a double-blind randomized control trial of intermittent preventive therapy during pregnancy in Uganda. Women were enrolled from 12 to 20 weeks gestation and followed through delivery. Exposure to malaria parasites was defined as symptomatic (fever with positive blood smear) or asymptomatic (based on molecular detection of parasitaemia done routinely every 4 weeks). The primary outcome was placental malaria diagnosed by histopathology, placental blood smear, and/or placental blood loop-mediated isothermal amplification. Multivariate analyses were performed using logistic regression models. Subgroup analysis was performed based on the presence of symptomatic malaria, gravidity, and timing of infection.

**Results:**

Of the 228 patients with documented maternal infection with malaria parasites during pregnancy, 101 (44.3%) had placental malaria. Primigravidity was strongly associated with placental malaria (aOR 8.90, 95% CI 4.34–18.2, p < 0.001), and each episode of malaria was associated with over a twofold increase in placental malaria (aOR 2.35, 95% CI 1.69–3.26, p < 0.001). Among multigravid women, the odds of placental malaria increased by 14% with each advancing week of gestation at first documented infection (aOR 1.14, 95% CI 1.02–1.27, p = 0.02). When stratified by the presence of symptoms, primigravidity was only associated with placental malaria in asymptomatic women, who had a 12-fold increase in the odds of placental malaria (aOR 12.19, 95% CI 5.23–28.43, p < 0.001).

**Conclusions:**

Total number of *P. falciparum* infections in pregnancy is a significant predictor of placental malaria. The importance of timing of infection on the development of placental malaria varies based on gravidity. In primigravidas, earlier asymptomatic infections were more frequently identified in those with placental malaria, whereas in multigravidas, parasitaemias detected later in gestation were associated with placental malaria. Earlier initiation of an effective intermittent preventive therapy may help to prevent placental malaria and improve birth outcomes, particularly in primigravid women.

## Background

Infection with malaria parasites during pregnancy is associated with increased risk of maternal and neonatal morbidity and mortality. In sub-Saharan Africa, where an estimated 11 million pregnancies are affected by malaria infection [1], malaria is thought to cause up to 100,000 infant deaths each year, the majority of which are secondary to prematurity, low birth weight, and neonatal anaemia [2, 3]. A unique characteristic of *Plasmodium falciparum* is the ability of infected erythrocytes to sequester in the placenta, leading to inflammation and adverse birth outcomes [4]. Placental malaria has been associated with increased risks of adverse obstetric outcomes including maternal anaemia, preterm delivery, fetal growth restriction, low birth weight, and maternal and neonatal mortality [5–7].

Previous studies have shown that primigravidity is a risk factor for placental malaria and resulting obstetric morbidity [8, 9]. A prior analysis of this study cohort found that placental malaria was more likely in women with high malaria burden (defined as ≥ 2 episodes of symptomatic malaria or ≥ 50% positive loop-mediated isothermal amplification (LAMP) samples) and was associated with increased rates of preterm birth and a trend towards higher rates of small for gestational age (SGA) neonates [10]. However, little is known about whether the effect of gravidity on placental malaria varies by timing and symptomatology of *P. falciparum*. A better understanding of how these factors influence placental infection in primigravid *versus* multigravid women may allow for more targeted public health interventions to reduce the prevalence of placental malaria and improve obstetric outcomes in both groups. Therefore, the main objective of this study was to assess whether the impact of gravidity on placental malaria varies by the presence of symptoms and/or timing of infection.

## Methods

### Study procedures

This was a secondary analysis of data collected from a double-blind, randomized trial of intermittent preventive therapy during pregnancy in Tororo, Uganda, a rural district in southeastern Uganda with an estimated entomologic inoculation rate of 310 infectious bites per person-year [11]. Participants were enrolled from June to October 2014; the complete study protocol for the parent study has been described elsewhere [11]. Briefly, pregnant women were enrolled at 12–20 weeks gestation and randomized to preventive therapy with sulfadoxine-pyrimethamine given every 8 weeks, dihydroartemisinin–piperaquine given every 8 weeks, or dihydroartemisinin–piperaquine given every 4 weeks during pregnancy. All participants were at least 16 years of age and HIV-uninfected at the time of enrollment.

At study enrollment, participants were assessed with standardized history and physical exam including confirmation of gestational age (GA) by ultrasound. Routine visits were scheduled every 4 weeks and included collection of dried blood spots for loop-mediated isothermal amplification (LAMP). Study participant were encouraged to come the study clinic any time they were ill and if they presented with a documented fever (tympanic temperature ≥ 38.0 °C) or a reported history of fever within 24 h prior to presentation, peripheral blood was collected for a thick blood smear. If the thick smear was positive, the participant was diagnosed with symptomatic malaria and treated with artemether-lumefantrine. Because LAMP results were not available until after completion of the trial, women with asymptomatic parasitaemia did not receive treatment. For the purposes of this analysis, women who presented with fever, but later had a positive LAMP were considered to have asymptomatic parasitaemia.

Placental tissue for histopathology, placental blood for thick smears and LAMP were collected within 1 h of delivery. Women who delivered at home were visited by study staff as soon as possible after delivery for evaluation and collection of specimens. Women with evidence of any infection with malaria parasites during pregnancy, known birth outcomes, and complete placental analysis (including placental histopathology, placental blood microscopy, and placental blood LAMP) were included in this study. *Plasmodium falciparum* infection was defined as both symptomatic malaria (fever and positive thick smear) and/or asymptomatic parasitaemia (peripheral maternal parasitaemia by LAMP in the absence of symptoms). The primary outcome, placental malaria, was defined as any evidence of placental infection, including the presence of parasites or pigment by histopathology, parasites detected in placental blood by microscopy, or positive placental blood LAMP.

### Laboratory methods

Dried blood spot samples were tested for *P. falciparum* DNA using LAMP at enrollment and every 4 weeks through pregnancy, as previously described [12, 13]. Placental tissue samples were fixed with formalin and embedded in paraffin. They were then processed and examined by two independent readers using a standardized case record form for histological evidence of placental malaria [11]. Any discrepancy between the readers was resolved by a third reader. Blood smears were stained with 2% Giemsa and evaluated by two trained laboratory technicians. A smear was considered negative if no asexual parasites were detected in 100 high-powered fields. Any discrepancies were again resolved by a third reader.

### Clinical variables of interest

Demographic data including maternal age, gravidity, body mass index (BMI), GA at enrollment and delivery, possession of bed net at enrollment, wealth status, and intermittent preventive therapy treatment arm were collected. Wealth status was defined as lowest, middle, and highest tertiles at study enrollment based on a composite variable of land ownership and possession of several household items [11]. GA was established at enrollment by obstetric dating by last menstrual period and ultrasound confirmation per WHO guidelines [14]. Malaria infection during pregnancy was defined as both symptomatic malaria and asymptomatic parasitaemia as previously mentioned, and longitudinal data on timing of infection during pregnancy were collected.

Birth outcomes including GA at delivery, birthweight, and fetal and neonatal survival to hospital discharge were collected and reported in previous analysis of this data set [10]. Preterm delivery was defined as delivery at < 37 weeks GA. SGA was defined as birthweight < 10%ile for GA based on East African fetal weight standards [15] which have been shown to be more accurate than other international growth standards in diagnosing SGA in this population [10, 16, 17]. For twin gestations, outcomes were considered positive if the outcome was seen in at least one twin or placenta.

### Statistical methods

Data analyses were performed using Stata 14 (Stata Corp, College Station, TX). Wilcoxon Rank Sum was used to compare median values of continuous variables, all of which were non-normally distributed. Chi squared and Fisher exact tests were used to compare proportions. Multivariate binary logistic regression using placental malaria as the main outcome were performed and were adjusted for primigravidity, GA at study enrollment, and GA at the time of the first recorded infection in pregnancy. Variables were included in the multivariate model if they were significant in the univariate analysis. Multivariate analyses stratified by gravidity, symptomatology and GA at initial infection were performed to examine interactions between possible predictors. The decision to do stratified analyses was made based on the a priori hypothesis that the relationship between these predictors could not be fully explained by multivariate analysis alone. All p values were two-sided and a value of p < 0.05 was considered statistically significant.

## Results

### Study participants

**Fig. 1:**
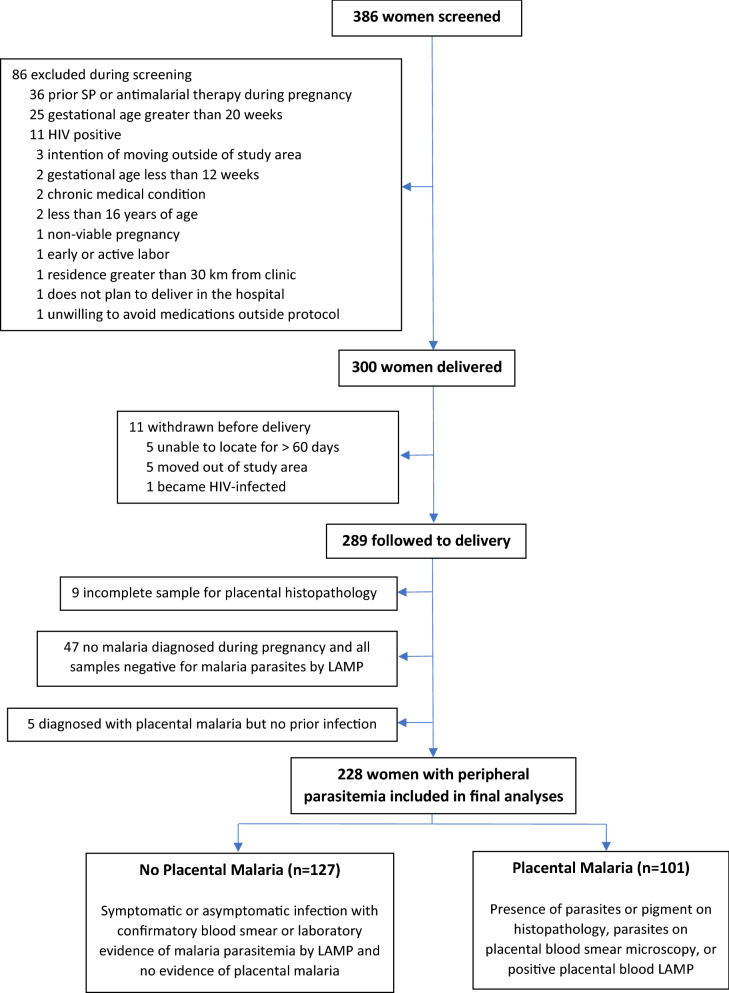
Flowchart of study participants. This figure displays the process for selecting study participants and the number of participants excluded in each category. *SP* sulfadoxine-pyrimethamine, *LAMP* loop-mediated isothermal amplification

**Fig. 2:**
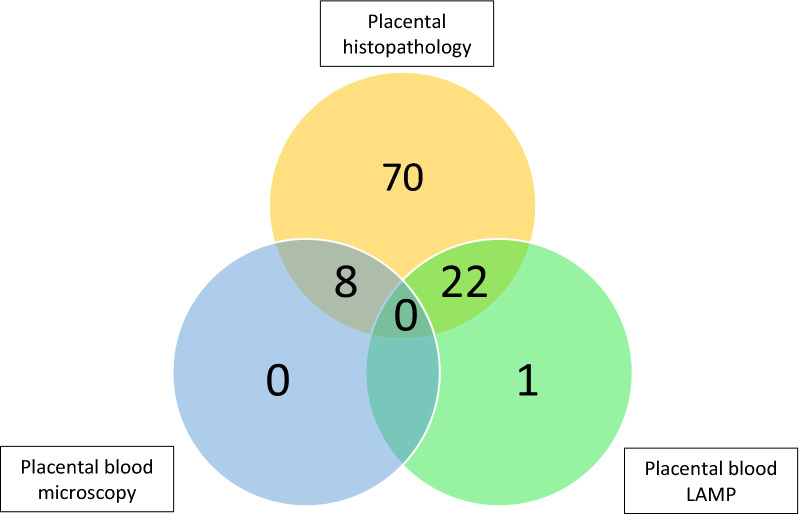
Number of placental malaria infections diagnosed by each method. The yellow circle holds women diagnosed by placental histopathology. The blue circle indicates diagnosis via placental blood microscopy, and the green circle represents diagnosis via placental blood LAMP

Of the 300 HIV-negative women enrolled in the parent trial, 289 (96.3%) were followed to delivery. Of these, nine (3.1%) were excluded for incomplete analyses for placental malaria including seven who did not have placental biopsies taken for histopathology and an additional two with missing results for placental blood analyses. Five women with placental malaria did not have documented evidence of parasitaemia prior to delivery and, given the inability to determine timing or number of infections, they were excluded from the analysis. Of the remaining 275 women, 228 (82.9%) had evidence of infection with malaria parasites during pregnancy and were included in the analyses. Of these, 127/228 (55.7%) did not have evidence of placental malaria and 101/228 (44.3%) had placental malaria (Fig. 1). The number of participants diagnosed with placental malaria via each of the three methods used are displayed in Fig. 2. Histopathology, which also detects past placental malaria, was the most sensitive of the three methods.

The median age of study participants was 21.1 years, and 38.2 percent were primigravid. Demographic characteristics and obstetrical outcomes of the study participants stratified by placental malaria status are presented in Table 1. Women with placental malaria were younger (19.0 years (17.9–21.3) vs. 22.7 (19.9–25.7); p < 0.01) (median (IQR)) and more likely to be primigravid (63.4% vs. 18.1%, p < 0.01). Those with placental malaria also enrolled (and therefore initiated intermittent preventive therapy) at a slightly later GA (15.3 weeks (14.0–17.6) vs. 14.7 (13.6–15.9) weeks, p < 0.01) and were significantly more likely to have received sulfadoxine-pyrimethamine treatment as compared to dihydroartemisinin–piperaquine (48.5% versus 31.5%, p ≤ 0.01). All of the included twin gestations (n = 4) had evidence of placental malaria in at least one placenta (Table 1). As previously reported [10], women with placental malaria delivered at an earlier GA (39.3 weeks (38.0–40.3) vs. 40.0 (39.0–40.9), p < 0.01) and were more likely to deliver preterm (< 37 weeks GA) (15.0% vs. 4.7%, p < 0.01) with correspondingly low birth weight (Table 1). Additionally, infants from pregnancies affected by placental malaria were more likely to be SGA (25.7% vs. 15.0%, p = 0.04). There were no significant differences in fetal or neonatal survival between the groups.

**Table 1:** Characteristics of study participants by placental malaria status

Variable	Placental malaria status in pregnancy		
	No placental malaria (n = 127)	Placental malaria (n = 101)	p value
Maternal age, years	22.7 (19.9–25.7)	19.0 (17.9–21.3)	*< 0.001*
Primigravid	23 (18.1)	64 (63.4)	*< 0.001*
BMI, kg/m^2^	20.6 (19.0–22.2)	21.0 (19.7–22.7)	0.08
Twin gestation^a^	0 (0.0)	4 (4.0)	*0.04*
GA at enrollment, weeks	14.7 (13.6–15.9)	15.3 (14.0–17.6)	*0.003*
Household wealth index			0.80
Lowest tertile	46 (36.2)	37 (36.6)	
Middle tertile	42 (33.1)	37 (36.6)	
Highest tertile	39 (30.7)	27 (26.7)	
Intermittent preventive therapy drug			*0.01*
Sulfadoxine-pyrimethamine	40 (31.5)	49 (48.5)s	
Dihydroartemisinin-piperaquine	87 (68.5)	52 (51.5)	
GA at delivery, weeks	40.0 (39.0–40.9)	39.3 (38.0–40.3)	*< 0.001*
Preterm birth < 37 weeks GA	6 (4.7)	15 (14.9)	*0.01*
Very preterm birth < 32 weeks GA	1 (0.8)	4 (4.0)	0.17
Low birth weight^b^	10 (7.9)	19 (18.8)	*0.01*
Small for gestational age^c^	19 (15.0)	26 (25.7)	*0.04*
Stillbirth	0 (0.0)	2 (2.0)	0.09
Neonatal demise^d^ (N = 224)	4 (3.2)	0 (0.0)	0.13

*BMI* body mass index, *GA* gestational age, *SGA* small for gestational age

Statistically significant p values (< 0.05) are indicated in *italics*

^a^Information missing for 1 patient

^b^Defined as birthweight < 2500 g

^c^Defined as birthweight < 10%ile for GA based on East African growth standards.^15^

^d^Data unavailable for four patients including two in the peripheral malaria group and 2 in the placental malaria group

Data are presented as median (interquartile range) or n (%). Wilcoxon Rank Sum and Chi squared or Fischer Exact tests were used to compare nonparametric continuous variables and proportions, respectively. Pregnancies with peripheral malaria only (without placental malaria) and pregnancies with placental malaria are the comparison groups for the p values

### Longitudinal measures of *P. falciparum* infection

**Fig. 3:**
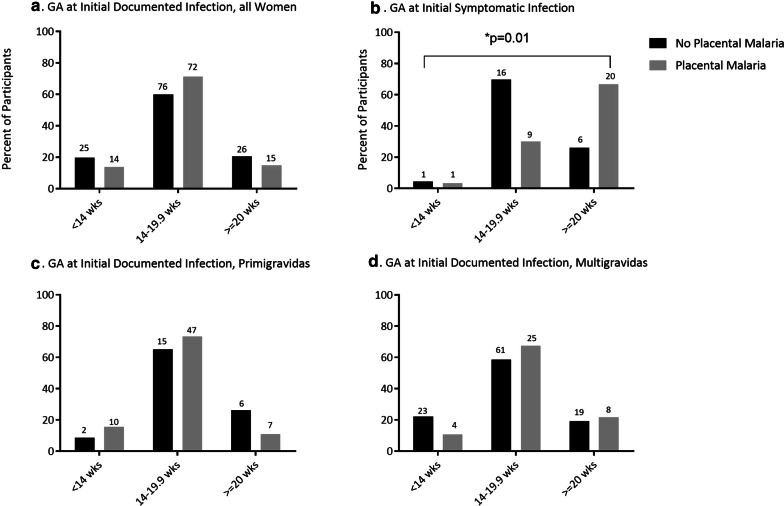
Timing of infection by malaria status and gravidity**. a** Shows the number (label) and percent of the total cohort (y-axis) of women whose initial documented infection occurred within each gestational age category separated for individuals with no placental malaria (black) and placental malaria (grey). **b** presents these data on initial symptomatic infection among symptomatic women. **c** Presents data for primigravid women only, while **d** presents data for multigravid women only. p-values are two-way and calculated with Chi squared analysis

Timing of initial documented infection and first episode of symptomatic malaria in women with and without placental malaria are depicted graphically in Fig. 3. Characteristics of infection are compared by placental malaria status in Table 2. Any symptomatic malaria at any time during pregnancy was more common in patients with placental malaria than those without placental malaria (29.7% vs. 18.1%, p = 0.04). Of those with at least one episode of symptomatic malaria, those with placental malaria had their first episode of symptomatic malaria at a later GA as compared to those without placental malaria (24.0 weeks (18.9–28.3) vs. 18.3 (16.1–20.0), p ≤ 0.01). GA at first documented infection (including symptomatic and asymptomatic) was not statistically different between the two groups (p = 0.15) (Table 2). Primigravidity, gestational age at enrollment, any symptomatic malaria during pregnancy and total number of times parasitaemia was detected in pregnancy were all controlled for in multivariate analyses because they were significant in univariate analyses. Gestational age at first documented infection was included in multivariate modeling rather than GA at first symptomatic infection to allow for examination of timing of infection without overfitting for symptomatology. In multivariate analyses, primigravidity, later GA at enrollment and increasing frequency of infections detected during pregnancy remained strongly associated with development of placental malaria (aOR 9.06, 95% CI 4.38–18.71, p < 0.001; aOR 1.24, 95% CI 1.02–1.50, p = 0.03; aOR 2.21, 95% CI 1.67–2.93, p < 0.001, respectively) (Table 3). As primigravid women are at increased risk of malaria infection, we also conducted the multivariate analysis without controlling for total number of infections in pregnancy in the model (Additional file 1: Table S1). Overall trends and statistical significance of findings for primigravidity and gestational age at enrollment were unchanged, and GA of first documented infection remains statistically insignificant. Any symptomatic malaria during pregnancy became statistically significant.

**Table 2:** Measures of malaria infection, by placental malaria status

Variable	Placental malaria status in pregnancy		
	No placental malaria (n = 127)	Placental malaria (n = 101)	p value
Positive LAMP at enrollment^a^	84 (66.1)	76 (75.3)	0.14
Number of times parasitaemia detected per pregnancy	2.0 (2.0–3.0)	3.0 (2.0–5.0)	*< 0.001*
Any symptomatic malaria during pregnancy	23 (18.1)	30 (29.7)	*0.04*
GA at first detection of symptomatic malaria, weeks	N = 23 18.3 (16.1–20.0)	N = 30 24.0 (18.9–28.3)	*0.01*
GA at initial documented parasitaemia, weeks	15.9 (14.3–17.9)	16.1 (14.7–18.6)	0.15

*GA* gestational age, *LAMP* loop mediated isothermal amplification

Data are presented as median (interquartile range) or n (%). Wilcoxon Rank Sum and Chi squared tests were used to compare nonparametric continuous variables and proportions, respectively

Statistically significant p values (< 0.05) are indicated in *italics*

^a^No patients had symptomatic malaria at the time of enrollment

**Table 3:** Predictors of placental malaria

Variable	aOR	95% CI	p value
Primigravidity	9.06	4.39–18.71	*< 0.001*
GA at enrollment, weeks	1.24	1.02–1.50	*0.03*
Any symptomatic malaria during pregnancy	1.88	0.82–4.32	0.14
GA of first documented infection, weeks	1.06	0.97–1.16	0.19
Total number of times parasitaemia detected in pregnancy	2.21	1.67–2.93	*< 0.001*

*GA* gestational age

Statistically significant p values (< 0.05) are indicated in *italics*

Multivariate binary logistic regression using placental malaria as the main outcome were performed and were adjusted for primigravidity, GA at study enrollment, any symptomatic malaria during pregnancy, GA of first documented infection, and total number of malaria infections (symptomatic and/or asymptomatic) in pregnancy. Variables were kept continuous where possible

### Subgroup analyses

**Fig. 4:**
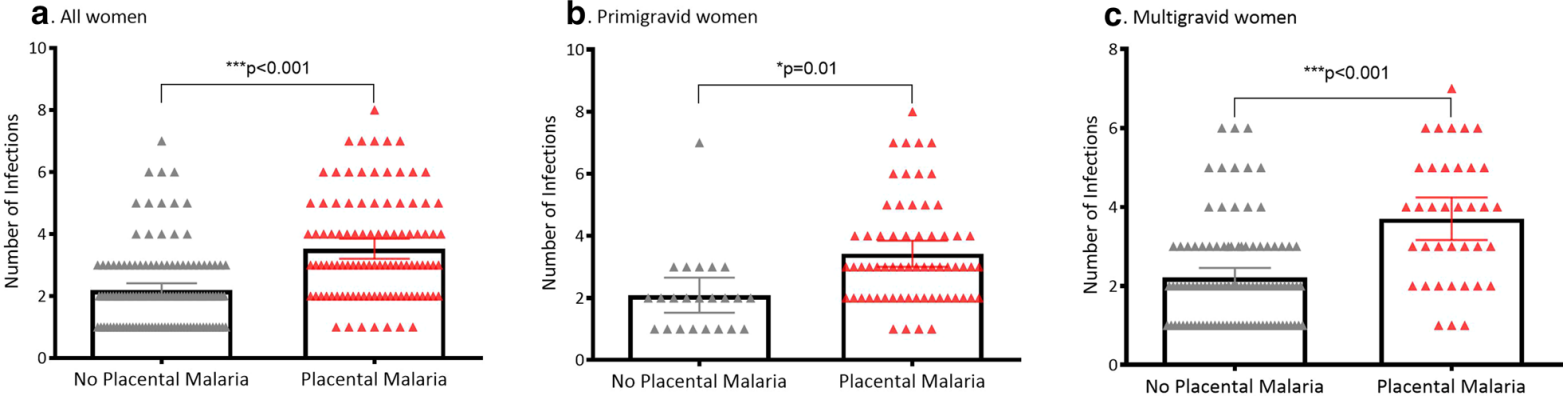
Frequency of infection by malaria status and gravidity. **a** Shows the frequency of malaria infection by malaria status (no placental malaria versus placental malaria) for all women. **b** Shows these data for primigravid women, while **c** shows these data for multigravida women. Each triangle represents one woman. Mean and 95% CI are represented with error bars. p values are two-way and calculated with Chi squared analysis

In primigravidas, each additional week of GA at enrollment was associated with an increase in the odds of placental malaria (aOR 1.84, 95% CI 1.21–2.78, p < 0.01), but this association was not seen in multigravidas. In multigravid women, each additional week of gestation at initial detected infection was associated with a 1.12-fold increase in odds of placental malaria (aOR 1.12, 95% CI 1.02–1.24, p = 0.02). This trend was not seen in primigravidas. Total number of times infection was detected in pregnancy remained strongly associated with placental malaria in both primigravid and multigravid women (Table 4 and Fig. 4).

**Table 4:** Predictors of placental malaria stratified by gravidity

Variable	Primigravid (N = 87)			Multigravid (N = 141)		
	aOR	95% CI	p value	aOR	95% CI	p value
GA at enrollment, weeks	1.84	1.21–2.78	*< 0.01*	1.12	0.88–1.44	0.36
Any symptomatic malaria in pregnancy	0.57	0.14–2.38	0.44	2.89	1.05–7.95	*0.04*
GA of first documented infection, weeks	0.82	0.67–1.01	0.06	1.12	1.02–1.24	*0.02*
Total number of times parasitaemia detected in pregnancy	2.70	1.44–5.06	*< 0.01*	2.30	1.65–3.23	*<0.001*

*GA* gestational age

Statistically significant p values (< 0.05) are indicated in *italics*

Multivariate binary logistic regression using placental malaria as the main outcome were performed and were adjusted for GA at study enrollment, GA of first documented infection, and total number of malaria infections (symptomatic and/or asymptomatic) in pregnancy. Variables were kept continuous where possible

Multivariate analyses stratified by presence of any symptomatic malaria or only asymptomatic parasitaemia was also performed. In women with only asymptomatic parasitaemia, primigravidity was associated with over a 12-fold increase in odds of developing placental malaria (aOR 12.07, 95% CI 5.24–27.81, p < 0.001), while there was no statistically significant association between primigravidity and placental malaria among those with any symptomatic malaria. In both groups, total number of infections in pregnancy remained highly associated with placental malaria (Table 5).

**Table 5:** Predictors of placental malaria stratified by presence of symptoms

Variable	Any symptomatic Malaria (N = 53)			Only asymptomatic parasitemia (N = 175)		
	aOR	95% CI	p value	aOR	95% CI	p value
Primigravid	2.63	0.54–12.76	0.23	12.07	5.24–27.81	*< 0.001*
GA at enrollment, weeks	1.36	0.89–2.08	0.15	1.26	1.00–1.58	*0.05*
GA of first documented infection, weeks	1.13	0.99–1.23	0.07	1.01	0.90–1.13	0.83
Total number of times parasitemia detected in pregnancy	2.43	1.37–4.30	**< ***0.01*	2.13	1.53–2.97	< 0.001

*GA* gestational age

Statistically significant p values (< 0.05) are indicated in *italics*

Multivariate binary logistic regression using placental malaria as the main outcome were performed and were adjusted for GA at study enrollment, GA of first documented infection, and total number of malaria infections (symptomatic and/or asymptomatic) in pregnancy. Variables were kept continuous where possible

Lastly, multivariate analyses were stratified by GA at initial documented infection. These categories were selected based on timing of enrollment in the study and subsequent testing: < 14 weeks (enrollment in first trimester), 14–19.9 weeks (enrollment in second trimester), and ≥ 20 weeks gestation (infection after study enrollment). Primigravidity was strongly associated with placental malaria in women whose initial documented infection occurred prior to 14 weeks (aOR 29.30, 95% CI 3.95–217.5, p = 0.001), as well as in women whose first infection occurred between 14 and 19.9 weeks (aOR 10.63, 95% CI 4.12–27.42, p < 0.001). Primigravidity was not a significant predictor of placental malaria in women whose initial infection occurred at or beyond 20 weeks gestation. Total number of times infection was detected in pregnancy, however, was strongly associated with placental malaria in women whose first infection occurred at or beyond 20 weeks (aOR 5.61, 95% CI 1.59–19.78, p < 0.01) as well as in women whose first infection occurred between 14 and 20 weeks gestation (aOR 2.63, 95% CI 1.80–3.87, p < 0.001) (Table 6).

**Table 6:** Predictors of placental malaria stratified by gestational age at initial documented infection

Variable	< 14 weeks (N = 39)			14–19.9 weeks (N = 148)			> = 20 weeks (N = 41)		
	aOR	95% CI	p value	aOR	95% CI	p value	aOR	95% CI	p value
Primigravid	29.30	3.95–217.5	*0.001*	10.63	4.12–27.42	*< 0.001*	6.02	0.79–46.10	0.08
GA at enrollment, weeks	0.78	0.14–4.33	0.78	1.58	1.19–2.09	*0.002*	1.14	0.77–1.67	0.51
Any symptomatic malaria in pregnancy	1.44	0.12–16.71	0.77	1.92	0.67–5.52	0.23	9.90	0.96–102.48	0.06
Total number of times parasitaemia detected in pregnancy	1.22	0.65–2.30	0.54	2.63	1.80–3.87	*< 0.001*	5.61	1.59–19.78	*< 0.01*

GA gestational age

Statistically significant p values (< 0.05) are indicated in *italics*

Multivariate binary logistic regression using placental malaria as the main outcome were performed and were adjusted for GA at study enrollment, GA of first documented infection, and total number of malaria infections (symptomatic and/or asymptomatic) in pregnancy. Variables were kept continuous where possible

## Discussion

### Principal findings

This study finds that the impact of gravidity on development of placental malaria varies by symptomatology and timing of *P. falciparum* infection. In women with only asymptomatic parasitaemia, primigravidity was the strongest predictor of placental malaria. In contrast, among women with symptomatic malaria, placental malaria was not significantly associated with gravidity. In regards to timing of infection, the magnitude of the association between primigravidity and the odds of placental malaria increased the earlier infection with malaria parasites was first detected, while multigravidity was associated with higher odds of placental malaria when infection occurred later in pregnancy. Of note, because the study population is women infected with malaria in pregnancy, the odds ratios reported in this study do not reflect odds of placental malaria in the general population, but rather odds of developing placental malaria if infected with *P. falciparum* in pregnancy.

When stratified by maternal gravidity, there were differences in the impact of timing of initial *P. falciparum* infection on the development of placental malaria. In multigravid women, later GA at initial infection is more strongly associated with placental malaria, while this is not observed in primigravid women. This suggests that much of the increased risk for primigravid women lies in early infection, which is not the case in multigravidas. This is consistent with the finding that later enrollment (and initiation of intermittent preventive therapy) increased odds of placental malaria in primigravid women but not in multigravid women.

This is the first study to explore the relationships between gravidity, symptomatology and timing of infection and the development of placental malaria. Prior literature on how the presence of malaria symptoms during pregnancy affects development of placental malaria is sparse. However, one cross sectional study of nulliparous Nigerian women found that more than 60% of asymptomatic women had evidence of placental parasitaemia at delivery, demonstrating that even asymptomatic infection has the potential to significantly impact obstetric outcomes [18]. This study adds to the current literature by highlighting that primigravid women are particularly vulnerable to placental malaria even when malaria infection remains clinically asymptomatic.

### Clinical implications

Previous studies exploring the impact of timing of malaria infection during pregnancy have been mixed, and few prior studies have examined the relationship between timing of infection in pregnancy and development of placental malaria despite the well-established association of placental malaria with increased adverse obstetric outcomes [10, 19, 20]. Several studies have found increased rates of SGA [21], decreased birthweight [22, 23], and decreased fetal growth velocity [24] with early malaria infection while others have found more significant detrimental effects on fetal growth with infection in the second or third trimesters [25] and/or at delivery [26, 27].

Additionally, several studies found a negative impact on fetal growth with both early and late infection [28–31]. Malaria infection in the third trimester and/or at delivery has also been associated with increased risk of preterm birth [21, 30]. Heterogeneity in the study designs, population demographics, surveillance protocols, diagnostic and treatment methodologies, and regional malaria characteristics may explain at least some of the discrepancies in these studies. Recently, an analysis of a separate cohort of women in Uganda found that burden of infection and timing of parasitaemia impact risk for placental malaria, which is consistent with the findings [32]. This study suggests that the importance of timing of infection on the development of placental malaria may vary based on gravidity. This finding may further explain why prior literature has painted an unclear picture of the impact of timing of infection on placental malaria.

Taken together, the results of this study have a number of implications for public health strategies going forward. Interventions aimed at preventing, identifying, and treating asymptomatic infections are likely needed to decrease placental malaria in primigravid women, whereas the magnitude of benefit in multigravid women may be less. Particular attention should be placed on preventing and treating early infections, both symptomatic malaria episodes as well as asymptomatic parasitaemia, in primigravid women. A challenge will be engaging women early in prenatal care. Finally, interventions aimed at reducing the overall number of *P. falciparum* infections during pregnancy are likely to benefit all pregnant women.

### Research implications

It is important to note that the LAMP results were not known until after delivery, and so did not impact clinical care. Future studies investigating the impact of treating based on prenatal surveillance for asymptomatic infections may improve outcomes, particularly in primigravid women who are of highest risk for adverse outcomes. Also, the effect of parasite density during pregnancy cannot be evaluated with LAMP, which is reported as positive or negative detection of parasite nucleic acid in the sample. These are important areas of future study.

### Strengths and limitations

There were many important strengths of this study including the longitudinal, prospective design, sonographically confirmed pregnancy dating, and rigorous screening protocol for both symptomatic and asymptomatic malaria infection throughout pregnancy. Additionally, examination of the placentas for evidence of malaria was undertaken by three different methods: placental blood smear, placental blood LAMP, and histopathology. Although the aforementioned strategy for placental analyses allowed for detection of microscopic as well as submicroscopic placental infection, histopathology was the most comprehensive method for diagnosis, as it also assessed for past placental malaria. Placental blood LAMP detected one additional case, but the overall benefit is unknown at this time.

There are several limitations of the study to acknowledge. Firstly, patients were enrolled between 12 and 20 weeks gestation, so details of malaria exposure in the first trimester are not known. The high proportion of patients with positive LAMP at enrollment, however, suggests widespread early exposure. Additionally, although monthly LAMP screening was performed throughout pregnancy, some malaria infections—particularly asymptomatic infections—that occurred between screening points may not have been detected. Our ability to distinguish persistent from recurrent infections as well as more definitively ascertain the timing of placental infection was also limited as parasite genotyping studies were not performed. Moreover, other obstetric comorbidities that may affect the rate of preterm birth and SGA, such as preeclampsia, bleeding or placental abruption, tobacco use, and infection were not consistently recorded.

## Conclusions

Total number of *P. falciparum* infections in pregnancy is a significant predictor of placental malaria regardless of gravidity, symptomatology, or timing of *P. falciparum* infection in pregnancy. The influence of gravidity on development of placental malaria depends on symptomatology and timing of infection. Earlier initiation of an effective intermittent preventive therapy treatment may be needed to prevent placental malaria and improve birth outcomes, particularly in primigravid women.

## Supplementary information

**Additional file 1: Table S1.** Multivariate analyses presented without controlling for total number of times parasitemia detected in pregnancy.

## Data Availability

The datasets generated and/or analysed during the current study are not publicly available due to patient privacy concerns but are available from the corresponding author on reasonable request.
