# Supplementary material for: The impact of gravidity, symptomatology and timing of infection on placental malaria
Source: Malar J. 2020 Jun 24;19:227. doi: 10.1186/s12936-020-03297-3 (PMC7315526; doi:10.1186/s12936-020-03297-3)
Supplement: Supplementary file 1 — Additional file 1: Table S1. Multivariate analyses presented without controlling for total number of times parasitemia detected in pregnancy. [file 12936_2020_3297_MOESM1_ESM.docx]

**Additional File: Table S1.**

| **Variable** | **aOR** | **95% CI** | **P value** |
| --- | --- | --- | --- |
| Primigravidity | 7.35 | 3.92-13.78 | **<0.001** |
| GA at enrollment, wks | 1.22 | 1.03-1.45 | **0.02** |
| Any symptomatic malaria during pregnancy | 2.34 | 1.14-4.79 | **0.02** |
| GA of first documented infection, wks | 0.97 | 0.89-1.04 | 0.44 |

GA = gestational age. Multivariate binary logistic regression using placental malaria as the main outcome were performed and were adjusted for primigravidity, GA at study enrollment, any symptomatic malaria during pregnancy, and GA of first documented infection. Variables were kept continuous where possible.
